# Focus on the prophylaxis, epidemiology and therapy of methicillin-resistant *Staphylococcus aureus* surgical site infections and a position paper on associated risk factors: the perspective of an Italian group of surgeons

**DOI:** 10.1186/s13017-016-0086-1

**Published:** 2016-06-14

**Authors:** G. Sganga, C. Tascini, E. Sozio, M. Carlini, P. Chirletti, F. Cortese, R. Gattuso, P. Granone, C. Pempinello, M. Sartelli, S. Colizza

**Affiliations:** Istituto Clinica Chirurgica, Divisione Chirurgia Generale e Trapianti d’Organo, Università Cattolica del Sacro Cuore, Fondazione Policlinico Universitario Agostino Gemelli, Rome, Italy; U.O. Malattie Infettive, Azienda Ospedaliera Universitaria Pisana, Pisa, Italy; U.O. Medicina d’Urgenza Universitaria, Azienda Ospedaliera Universitaria Pisana, Pisa, Italy; Chirurgia generale Ospedale Sant’Eugenio di Roma, Rome, Italy; Dipartimento di Chirurgia Università La Sapienza, Policlinico Umberto I, Rome, Italy; UOC Chirurgia di Urgenza Ospedale San Filippo Neri, Rome, Italy; Dipartimento di Chirurgia Generale e Trapianti d’Organo, UOC Chirurgia Vascolare, Università La Sapienza, Policlinico Umberto I, Rome, Italy; Istituto Patologia Chirurgia, Unità Operativa Complessa Chirurgia Toracica, Università Cattolica del Sacro Cuore, Fondazione Policlinico Universitario Agostino Gemelli, Rome, Italy; Ortopedia e Traumatologia dell’Ospedale S. Gennaro ASL Napoli 1 Centro, Naples, Italy; U.O Chirurgia Generale Ospedale di Macerata, Macerata, Italy; Master Sepsi in Chirurgia, Università Cattolica, Rome, Italy

**Keywords:** Surgical site infection, MRSA, Dalbavancin

## Abstract

**Background:**

The aim of this research was to study the epidemiology, microbiology, prophylaxis, and antibiotic therapy of surgical site infections (SSIs), especially those caused by methicillin-resistant *Staphylococcus aureus* (MRSA), and identify the risk factors for these infections. In Italy SSIs occur in about 5 % of all surgical procedures. They are predominantly caused by staphylococci, and 30 % of them are diagnosed after discharge. In every surgical specialty there are specific procedures more associated with SSIs.

**Methods:**

The authors conducted a systematic review of the literature on SSIs, especially MRSA infections, and used the Delphi method to identify risk factors for these resistant infections.

**Results:**

Risk factors associated with MRSA SSIs identified by the Delphi method were: patients from long-term care facilities, recent hospitalization (within the preceding 30 days), Charlson score > 5 points, chronic obstructive pulmonary disease and thoracic surgery, antibiotic therapy with beta-lactams (especially cephalosporins and carbapenem) and/or quinolones in the preceding 30 days, age 75 years or older, current duration of hospitalization >16 days, and surgery with prothesis implantation. Protective factors were adequate antibiotic prophylaxis, laparoscopic surgery and the presence of an active, in-hospital surveillance program for the control of infections. MRSA therapy, especially with agents that enable the patient’s rapid discharge from hospital is described.

**Conclusion:**

The prevention, identification and treatment of SSIs, especially those caused by MRSA, should be implemented in surgical units in order to improve clinical and economic outcomes.

## Background

Surgical site infections (SSIs) remain a major clinical problem in terms of morbidity, mortality, time spent in hospital and overall direct and indirect costs [1–3]. Despite progress in their prevention, SSIs remain one of the most common adverse events in hospitals, accounting for 11 % to 26 % of all healthcare-associated infections [4]. Surgical patients can develop several post-operative infections; wound infections, are common causes of post-operative morbidity and prolonged hospitalization. SSIs increase the total hospital bill by an additional 10–20 % [5], lead to 80,000 deaths and are associated with an annual treatment cost of two billion US dollars [6]. *S. aureus* SSIs can be life-threatening, being associated with a mortality rate of 5 %, more than 2 extra weeks of time spent in hospital and around an extra cost of 50,000 US dollars [7].

Nearly 60 % of SSIs are diagnosed after discharge from the hospital. This percentage is rising as the post-operative stay in hospital is getting shorter and the number of 1-day surgical procedures is increasing over time [8]. The Diagnosis Related-Group (DRG) system underestimates the rate of SSIs because of the very early discharge of surgical patients. The exact incidence of SSIs is, therefore, difficult to determine. According to current literature, active SSI surveillance is useful in reducing the incidence of SSIs by surveillance-induced infection control efforts [9]. When any prosthesis is implanted into the body during general surgery, cardiac surgery, orthopaedics, etc., beside *S. aureus*, Coagulase-negative Staphylococci, especially *S. epidermidis*, may be the cause of severe infections that can necessitate removal of the prosthesis.

### Classification of surgical site infections

The identification of SSIs involves interpretation of clinical and laboratory findings. The Centers for Disease Control and Prevention’s (CDC) National Nosocomial Infections Surveillance system has developed standardized surveillance criteria for defining SSIs [10]. According to these criteria, SSIs are classified as being either incisional or organ/space. Incisional SSIs are further divided into those involving only skin and subcutaneous tissue (superficial incisional SSIs) and those involving deeper soft tissues of the incision (deep incisional SSIs).

#### Superficial incisional surgical site infections

The infection occurs within 30 days after the operation and involves only the skin or subcutaneous tissue at the incision and at least one of the following:

1. purulent drainage, with or without laboratory confirmation, from the superficial incision;
2. organisms isolated from an aseptically obtained culture of fluid or tissue from the superficial incision;
3. at least one of the following signs or symptoms of infection: pain or tenderness, localized swelling, redness, or heat; the site is deliberately opened by the surgeon, unless ithe incision is culture-negative.

#### Deep incisional surgical site infections

The infection occurs within 30 days after the operation if no implant is left in place or within 1 year if an implant is left in the body and the infection appears to be related to the operation and involves deep soft tissues (e.g., fascial and muscle layers) at the incision site and at least one of the following:

1. purulent drainage from the deep incision but not from the organ/space component of the surgical site;
2. a deep incision spontaneously dehisces or is deliberately opened by a surgeon when the patient has at least one of the following signs or symptoms: fever (>38 °C), localized pain, or tenderness, unless the site is culture-negative.
3. an abscess or other evidence of infection involving the deep incision is found on direct examination, during reoperation, or by histopathological or radiological examination.

#### Organ/space surgical site infections

The infection occurs within 30 days after the operation if no implant is left in place or within 1 year if an implant is left in the body and the infection appears to be related to the operation and involves any part of the anatomy (e.g., organs or spaces), other than the incision, which was opened or manipulated during an operation and at least one of the following:

1. purulent drainage from a drain that is placed through a stab wound into the organ/space.
2. organisms isolated from an aseptically obtained culture of fluid or tissue in the organ/space.
3. an abscess or other evidence of infection involving the organ/space that is found on direct examination, during reoperation, or by histopathological or radiological examination.

Recently, the Food and Drug Administration (FDA) proposed a new classification of skin and soft tissue infections, namely acute bacterial skin and skin structure infections (ABSSSIs), incorporating erysipelas, cellulitis, major subcutaneous abscesses and wound infections including SSIs. An ABSSSI is a bacterial infection of the skin with a lesion area of at least 75 cm^2^, measured by redness, edema, or induration [11]. ABSSSIs represent a significant burden for the health-care system, with increasing incidence and severity in recent years, and they have become a challenging medical problem associated with high direct and indirect costs to both the medical system and society. Among the various ABSSSIs, SSIs caused by MRSA may have an important impact on hospital budgets and on patients’ health. The rationale for introducing this terminology was to provide a consistent means of identifying infections for which a reliable drug treatment effect can be estimated and of correctly stratifying patients with this kind of infection included in clinical trials.

### Microbiological epidemiology

According to the National Nosocomial Infection Surveillance system reports, Gram-positive cocci (particularly *S. aureus*, coagulase-negative staphylococci and *Enterococcus* spp.), followed by *Escherichia coli*, *Pseudomonas aeruginosa* and *Enterobacter* spp., are the most commonly encountered pathogens in SSIs [12]. In most cases, the source of pathogens is the endogenous flora of the patient’s skin, mucous membranes, or hollow viscera [13]. When mucous membranes or the skin are incised, the exposed tissues are at risk of contamination by endogenous flora [14]. These micro-organisms are usually aerobic Gram-positive cocci (e.g., staphylococci), but may include fecal flora (e.g., anaerobic bacteria and Gram-negative aerobes) when incisions are made near the perineum or groin. Exogenous sources of SSI pathogens include surgical personnel (especially members of the surgical team), the operating room environment (including air), and all equipment, instruments, and materials brought to the sterile field during an operation. Exogenous flora consists primarily of aerobes, especially Gram-positive organisms (e.g., staphylococci and streptococci) [15].

Table 1 presents the more frequent pathogens according to the surgical procedure.

**Table 1:** More frequent pathogens according to the surgical procedure

Type of surgery	Likely Pathogens
Placement of all grafts, prostheses, or implants	*S. aureus*, coagulase negative staphylococci
Cardiac	*S. aureus*, coagulase negative staphylococci
Neurosurgery	*S. aureus*, coagulase negative staphylococci
Breast	*S. aureus*, coagulase negative staphylococci
Ophthalmic (limited data, however, commonly used in procedures such as anterior segment resection, vitrectomy, and scleral buckles)	*S. aureus*, coagulase negative staphylococci, streptococci, gram-negative bacilli
Orthopedic (Total joint replacement, closed fractured/use of nails, bone plates, other internal fixation device, functional repair without implant/device trauma)	*S. aureus*, coagulase negative staphylococci, gram-negative bacilli
Non-cardiac thoracic (lobectomy, pneumonectomy, wedge resection, other non-cardiac mediastinal procedures) Closed tube thoracotomy	*S. aureus*, coagulase negative staphylococci*, S. pneumoniae*, gram-negative bacilli
Vascular	*S. aureus*, coagulase negative staphylococci
Appendectomy	Gram-negative bacilli, anaerobes
Biliary tract	Gram-negative bacilli, anaerobes
Colorectal	Gram-negative bacilli, anaerobes
Gastroduodenal	Gram-negative bacilli, streptococci, oropharyngeal anaerobes (e.g. peptostreptococci)
Head and neck (majorly procedures with incision through oropharyngeal mucosa)	*S. aureus*, streptococci, oropharyngeal anaerobes (e.g. peptostreptococci)
Obstetric and gynecologic	Gram-negative bacilli, enterococci, group B streptococci, anaerobes
Urologic	Gram-negative bacilli

### Methicillin-resistant *Staphylococcus aureus*

Methicillin-resistant *Staphylococcus aureus (*MRSA) is a versatile and dangerous bacterial pathogen, combining virulence, antibiotic resistance, and survival fitness [16]. Its spread is facilitated by cross-transmission through health-care workers’ hands and selection pressure exercised by broad-spectrum antibiotic treatment. MRSA infections represent a substantial burden, because they increase treatment costs and can cause excess morbidity and mortality. Since MRSA carriers without symptomatic infection are an important reservoir and source of spread of infections, risk profiles to identify patients at high risk of carrying MRSA might improve prevention of MRSA infections [17].

The control of SSIs within the hospital is important, as is identifying patients at risk of being colonized and subsequently infected by multi-drug resistant microorganisms such as MRSA; surveillance itself, even without any specific intervention, has been associated with a reduction in the incidence of SSIs, which is another reason to recommend implementation of surveillance systems [18].

According to the new ABSSSI classification, erysipelas and cellulitis are mostly due to Gram-positive organisms whereas SSIs and subcutaneous abscesses are due to mixed flora including Gram-positive microbes.

### The epidemiology of surgical site infections in Italy

In Italy the incidence rate of SSIs might be around 5 % (Table 2). Hospitals without a surveillance system that report rates less than 5 % probably underestimate the problem. In fact many SSIs are diagnosed more than 30 days after surgery, when the patient has already been discharged from hospital. The overall incidence rate of SSIs in Italy in the past was higher, ranging from 5.4 % up to 12.8 % [19, 20]. In Europe, considering only general, gynecological (including Cesarean sections), and vascular surgery, SSIs rates are higher and range from 6.34 % to 14.8 % [21].

**Table 2:** SSI epidemiology in Italy

Authors	Country	Period	Number of surgeries	SSIs
Petrosillo et al.	Italy, multicenter	2002, Prospectively for one month in 48 hospitals	4.665	241 (5,2 %)
Marchi et al.	Italy, multicenter	2009–2001, retrospective, 355 surgical departments	60.460	1.628 (2,6 %)
Fiorio et al.	Italy, multicenter	2002, Prospectively for one month in 48 hospitals, 32 surgical departments	2.972	158 (5,2 %)

Petrosillo et al. reported that SSIs occurred in 5.2 % of 4,665 patients. Of these SSIs, 148 (61.4 %) developed during the patients’ stay in hospital, and 93 (38.6 %) within 30 days after discharge. Of the 148 in-hospital SSIs, 87, 37, and 24 were classified as superficial, deep, and organ/space, respectively. Post-discharge SSIs observed at medical follow-up were all superficial [21].

This study also produced information on post-discharge SSIs; the authors found that 38.6 % of SSIs were diagnosed after the patients’ discharge from hospital; this rate is comparable to the 34.8 % reported by Fiorio et al. [22] in Italian general surgery in-patients, although other similar surveillance studies [23] found considerably higher post-discharge SSIs rates, ranging from 34.8 % to 60 %.

Marchi et al. reported data on non-prosthetic surgery from the Italian SSI surveillance program for the period 2009 to 2011. In the 355 surgical wards included in the study, 60,460 operations were recorded. SSIs were observed in 1,628 cases (2.6 %) and 60 % of the infections were diagnosed through 30-day post-discharge surveillance. Operations performed in hospitals with at least 2 years of surveillance showed a 29 % lower risk of SSIs, confirming that surveillance is a protective factor for preventing SSIs [24].

Table 3 reports the probability of developing a post-discharge SSI according to type of surgery performed. Used vascular surgery as the benchmark (0 % of infections after discharge) each number represents the probability of a post-discharge diagnosis of a SSI.

**Table 3:** Probability to have a post-discharge SSIs according to type of surgery performed

Surgery	Ratio between post-discharge SSIs and in-hospital SSIs
Vascular surgery	0
Breast surgery	3,4
Cesarean section	2,5
Cholecistectomy	1,2
Gastric surgery	0,4
Appendectomy	0,2
Colon surgery	0,1
Hernia repair	1,4
Abdominal hysterectomy	0,6
Vaginal hysterectomy	1

### Specific types of surgical procedures and surgical site infections

#### Hernia and abdominal wall surgery

Although this type of surgery is “clean and easy” to perform, the usual application of meshes might expose patients to a higher risk of SSIs.

According to the Italian Society of Hernia and Abdominal Wall Surgery, 210,000 surgical procedures per year are performed on the abdominal wall in Italy; this kind of procedure is, therefore, the most common type in Italy. These procedures comprise 170,000 inguinal hernia repairs per year, and 40,000 parietal hernia repairs, with this latter group including 20,000 incisional hernias. Almost all of these procedures are performed using meshes in order to reduce the incidence of recurrence and to achieve closure of the abdominal wall defect avoiding abdominal hypertension and respiratory complications. The use of meshes may increase the risk of SSIs caused by difficult-to-treat microorganisms such as *S. aureus*, coagulase-negative staphylococci and multidrug-resistant nosocomial Gram-negative organism [25], leading to infection of the mesh itself.

Despite hernia repairs being classified as “clean” operations, the use of synthetic material has been associated with a theoretical increase in infectious risk. Although routine use of prophylactic antibiotics is not recommended for open, non-implant herniorrhaphy, there is little direct clinical evidence on which to base recommendations when an implantable mesh is used. No SSIs occurred following 120 mesh herniorrhaphies performed without routine antibiotic administration [26]; however, the incidence of SSIs varies, depending on the different case series, ranging from 1–2 % to 3–4 %.

Mesh infection, although infrequent, is a devastating complication of mesh hernioplasties. The crude mesh infection rate was 5 % in a recent meta-analysis. Statistically significant risk factors were smoking, American Society of Anesthesiologists (ASA) score C3 and emergency operation; mesh infections were also significantly correlated with the patients’ age and the duration of the hernioplasty. A trend toward higher mesh infection rates was observed in obese patients. The usual causative organisms associated with cases of mesh infection are *Staphylococcus* spp., especially *S. aureus*, *Streptococcus* spp. (including group B streptococci), Gram-negative bacteria (mainly Enterobacteriaceae), and anaerobic bacteria (including *Peptostreptococcus* spp.) [25]. In a study of mesh-related infections following incisional herniorrhaphy, 63 % of the microorganisms isolated were MRSA [27].

Table 4 shows the microbiology of prosthetic infection after hernia or ventral mesh repair.

**Table 4:** Microbiology of prosthetic infection (1 day- > 1 year) after haernia or ventral mesh repair

Microbiology of prosthetic infection (1 day- > 1 year) after haernia or ventral mesh repair	
*Staphylococcus aureus*	50–60 %
*Staphylococcus spp*	10–20 %
*Enterococcus spp*	10–20 %
Gram-neg	5–10 %
Pseudomonas, *Streptococcus spp*, *Mycobacteria spp*, mixed flora	rare

Theoretically, surgical treatment of infected mesh hernioplasties requires removal of the implant and then implantation of a new mesh; conservative treatments, such as drainage or sinus resection, are usually unsuccessful. Tolino et al. reported on 51 operations to treat 32 patients with mesh infections: removal of the mesh, recurrence and bowel fistulas were described [28].

Every year in Italy there are probably about 1,700 infections of inguinal hernioplasties and 4,000 infections of ventral hernioplasties (for %1 and 10 % rates of infective complications, respectively). The role of *S. aureus* and MRSA in the etiology of these complications is very strong [28, 29].

#### Vascular surgery

The incidence of SSIs at the groin after vascular procedures ranges from 3 % to 44 % [30]. Factors contributing to the increased incidence of SSIs in this subset of patients include disruption of the lymphatic system, proximity of the surgical site to the perineum and external urinary organs, and prosthetic graft placement. It must be emphasized that the groin area contains a substantial number of lymph nodes which have a priority role in draining the entire lower limb and an incorrect surgical technique often leads to lymphorrhea with a subsequent SSI. A SSI following vascular surgery is a complication that may lead not only to healing problems, but also to limb loss and the risk of death. Furthermore, it is assumed to be a risk factor for deeper infections extending to the graft, which are associated with catastrophic consequences: a death rate between 15 % and 75 % and a major amputation rate of up to 70 %. Even when treatment is successful, the morbidity associated with vascular graft infection is considerable, the outcomes often being worse than the natural history of the vascular condition that led to implantation of the graft.

A SSI always implies prolongation of the time spent in hospital, higher hospital costs and greater morbidity for the patient. Groin incisions play a central role in various vascular operations. In addition to their traditional use in bypass procedures, endarterectomies and thromboembolectomies, more than 95 % of all percutaneous interventions are performed via the femoral access. The increasing prevalence of infections by multiresistant bacteria has been associated with worsened outcomes. Although the management of groin SSIs is well documented, there is little published information about the incidence and classification of these infections. Many authors consider that diabetes, as well as obesity, has a relatively minor role in initiating groin wound infections. Besides general aseptic measures, the only strategy that significantly reduces the incidence of SSIs is prophylactic systemic antibiotics, administered only preoperatively. There is no “gold standard” treatment of these infections. The initial treatment generally includes surgical debridement and antibiotics. Pharmacological therapy is very often prolonged for several weeks but with uncertain results. Secondary interventions have been attempted with limited success; such interventions include rotational flaps, wound vacuum-assisted closure, alginate dressing, graft excision and extra-anatomic or in situ bypass or patch replacement using autologous, rifampin-soaked or silver-impregnated materials.

#### Thoracic surgery

Thoracic surgery includes a wide variety of operations involving the lungs and pleural cavities, mediastinum, oesophagus and neck.

The most common surgical incisions in thoracic surgery are thoracotomies, cervicotomies, and thoraco-abdominal incisions; sternotomies are less common.

The overall incidence of SSIs in thoracic surgery has been estimated to be around 2–4 %.

Esophageal surgery, especially when the indication is cancer, is the operation most frequently complicated by SSIs, accounting for up to 15 % of cases due to radical dissection, difficult reconstruction, frequent concomitant colonization, and patients being at higher risk because of concomitant conditions (age, smoking, alcoholism, malnutrition, etc.).

The most common procedure, thoracotomy for lung cancer, has been estimated to be complicated by SSIs in only 2 % to 5 % of cases. However, this type of SSI can produce serious discomfort for the patient, due to the need for ongoing medication, pain, difficulty with breathing with the risk of developing more serious infections such as pneumonia, pleural empyema and mediastinitis.

Most recently mini-invasive thoracic approaches and video-assisted thoracoscopic surgery have reduced the overall wound infection rate to a mean of 1.7 % [31].

Deep sternal infections in cardiothoracic surgery, despite accounting only for 1–4 % cases, can be associated with ten-fold higher mortality rate.

An American study from 2002 showed that risk factors for deep and superficial chest SSIs after coronary artery bypass surgery differ, suggesting different etiopathogenesis. However, some of the risk factors for thoracic SSIs are well known, such as age, smoking, diabetes, obesity and, in particular, chronic obstructive pulmonary disease requiring pre-operative treatment with systemic antibiotics and nebulisers [32]. The need for neoadjuvant chemoradiotherapy in advanced cancer is another independent risk factor.

It seems obvious that all the risk factors for post-operative infections in such fragile patients become even more critical because the infections are caused by multi-resistant organisms. The main sources of multi-resistant infections are Gram-positive bacteria, particularly MRSA, but other sources, such as fungi, are becoming increasingly common.

#### Orthopedic surgery

Because orthopedic procedures are performed in a variety of inpatient and outpatient settings, increased vigilance, strict adherence to aseptic technique, attention to adequacy of reprocessing and management of intraoperative breaches of sterile technique are vitally important to ensure a safe and consistent standard of care.

According to the National Healthcare Safety Network report, which includes data from 2006 to 2008 in the USA, the total number of patients who develop SSIs following any type of orthopedic surgery is somewhere between 31,000 and 35,000 [33]. SSIs in orthopedic surgery prolong total hospital stay by a median of 2 weeks per patient, approximately double readmission rates, and increase health care costs by more than 300 %. Moreover, patients with SSIs have substantially greater physical limitations and significant reductions in their quality of life [33].

The rates of SSIs following orthopedic procedures appear to be increased when certain risk factors are present. Risk factors can be either patient modifiable or not modifiable and/or procedure-specific, in which case they may again be modifiable or not modifiable.

Hair removal, perioperative normothermia, preoperative skin preparation, nasal decolonization, health problems and situations predisposing the patient to infection, skin antisepsis, surgical hand antisepsis, antibiotic prophylaxis, air quality, double gloves, traffic pattern gowns and drapes, bone cement, and sterility assurance are key factors for controlling the risk of infections.

Although the use of antimicrobial sutures is not routine practice, the benefits of this strategy are becoming increasingly apparent [34]. Likewise, advances in antimicrobial coatings for implants, instruments, equipment and the environment may provide additional support to reach the goal of no SSIs. The practice of prescreening patients prior to surgery is recommended, as part of a comprehensive program to eliminate SSIs in orthopedic surgery, especially in cases involving an implantable device.

### Type of surgery and risk of surgical site infections

Attempts have been made to quantify the risk of SSIs following types of surgery other than operations to the abdominal wall..

De Lissovoy et al. performed a study with the aim of analyzing the surgical procedures associated with the highest rate of SSIs; they studied 723,490 discharges from surgery units in 2005, identifying SSIs from the presence of The International Classification of Diseases, Ninth Revision, Clinical Modification (ICD-9-CM) code 998.59. They divided surgical procedures into seven categories: neurological; cardiovascular, colorectal, skin, subcutaneous and breast; gastrointestinal, orthopedic and obstetric-gynecological. This made it possible to quantify the risk of SSIs according to the type of surgical procedure used.

Table 5 lists the five most frequent surgical procedures by category with respect to the incidence of SSIs.

**Table 5:** The 5 most frequent surgical procedures for categories with respect to the incidence of SSIs

Surgery category	Procedure	Percent of discharge	Percent with SSI
Neurologic	03.59 Spinal structure repair not elsewhere classified	1,34	2.53
	81.05 Other dorsolum fusion	0,27	2,29
	02.12 Brain meninges repair not elsewhere classified	0,75	2,25
	01.25 Other craniectomy	0,22	2,1
	01.53 Brain lobectomy	0,29	2,09
Cardiovascular	35.14: Open heart valvuloplasty of tricuspid valve without replacement	0,3	4,32
	35.84: Total correction of transposition of great vessels	0,35	4,09
	35.91: Interatrial venous return transposition	0,08	3,85
	37.22: Left heart cardiac catherization	0,17	3,74
	35.28: Replace tricuspid valve not elsewhere classified	0,09	3,7
Colorectal	46.74: Closure small bowel fistula not elsewhere classified	0,28	10,0
	45.93: Small-to-large bowel not elsewhere classified	0,55	9,24
	45.61: Multiple segmental small bowel excision	0,55	8,73
	46.20: Ileostomy not otherwise specified	0,19	8,59
	46.01: Small bowel exteriorization	0,84	6,34
Breast Skin and subcutaneous tissue	54.11: Exploratory laparotomy	0,5	2,5
	84.15: Below knee amputation not elsewhere classified	0,32	1,96
	40.11: Lymphatic structure biopsy	0,35	1,79
	85.45: Unilateral radical mastectomy	0,6	1,57
	54.30: Destruction abdominal wall lesion	0,42	1,49
Gastrointestinal	52.70: Radical pancreaticoduodenectomy	0,78	7,74
	43.70: Partial gastrectomy with jejunum anastomosis	0,89	5,63
	42.41: Partial esophagectomy	0,25	5,03
	43.99: Total gastrectomy not elsewhere classified	0,49	4,99
	86.22: Wound debridement	0,44	4,97
Orthopedic	80.05: Arthrotomy/prosthesis removal-hip	0,22	1,74
	79.85: Open reduction-hip dislocatation	0,64	1,19
	77.85: Partial ostectomy-femur	0,5	0,76
	77.25: Femoral wedge osteotomy	0,55	0,69
	80.15: Other arthrotomy-hip	0,88	0,66
Obstetric/ gynecologic	68.80: Pelvic evisceration	0,04	10,48
	68.40: Total abdominal hysterectomy	0,03	4,3
	40.54: Radical groin dissection	0,01	4,17
	71.50: Radical vulvectomy	0,2	3,42
	40.52: Radical dissection periaortic lymph node	0,08	2,3

### Antimicrobial prophylaxis

The prevention of SSIs is an important issue in surgery. Besides general strategies aimed at reducing the rate of SSIs (hair removal, asepsis of the operating room, glucose control, blood transfusions, and smoking cessation) antibiotic prophylaxis plays a pivotal role in reducing SSIs.

The degree of bacterial contamination during surgery, together with the ASA score and duration of surgery, define the appropriateness of antibiotic prophylaxis: surgical procedures are classified, as well-known, into clean, clean-contaminated, contaminated and dirty.

Dirty surgery should be treated as therapy of the infection involved (no prophylaxis).

Clean surgery usually does not need prophylaxis, unless comorbidities are present and/or prostheses are used.

The main indication for prophylaxis is, therefore, clean-contaminated or contaminated surgery. The purpose of antibiotic prophylaxis is only to avoid SSIs, not to prevent any other post-operative infectious complications (pneumonia, urinary tract infections, etc.).

The choice of antibiotic for prophylaxis should be directed towards the more probable pathogens for the particular type of surgery. Furthermore, antibiotics with a broad spectrum of action should not be used, because such these are the drugs of choice to treat an infection empirically.

Prophylaxis should be administered intravenously, half an hour before the start of surgery and should not be continued for more than 24 h.

Table 6 lists the recommended antibiotics for antimicrobial prophylaxis [35] according to the type of surgery, and the alternative drugs in case of allergy to penicillin and/or cephalosporins. It is clear that the most frequently used antibiotic is cefazolin alone, combined with metronidazole when anti-anaerobe coverage is required and with vancomycin in selected cases of cardiovascular, orthopedic or neurosurgery when devices and/or prostheses are used.

**Table 6:** The recommendations for antimicrobial prophylaxis [30]

Surgical category	Routine antibiotic prophylaxis	Penicillin or cephalosporin allergy
Cardiac	Cefazolin plus vancomycin (device only)	Vancomycin or Clindamycin plus gentamicin
Thoracic	Cefazolin	Vancomycin or Clindamycin
Colorectal	Cefazolin plus metronidazole	Clindamycin plus gentamicin
Otorlaryngology	Cefazolin alone or plus metronidazole	Clindamycin alone or plus ciprofloxacin
General surgery	Cefazolin	Clindamycin alone or plus gentamicin
Hepatobiliary (complicated)	Cefazolin	Vancomycin plus tobramycin
Neurosurgery	Cefazolin plus vancomycin (device only)	Vancomycin
Orthopedic	Cefazolin plus vancomycin (artroplasties only)	Vancomycin or Clindamycin
Obstetric/gynecologic	Cefazolin	Clindamycin
Vascular	Cefazolin plus vancomycin (syntetic graft only)	Vancomycin
Plastics, Reconstructive & Hand Surgery	Cefazolin	Clindamycin
Urology	Cefazolin	Ciprofloxacin alone or plus vancomycin

According to the guidelines, if the surgical procedure lasts more than 3–4 h, there is an indication to administer another dose of prophylactic antibiotic, during surgery. In the case of renal impairment, this interval should be modified according to the creatinine clearance.

### Therapy for methicillin-resistant *Staphylococcus aureus* infections

The guidelines of the Infectious Diseases Society of America recommend treatment with a beta-lactam or clindamycin for mild/moderate, non-purulent ABSSSIs and vancomycin plus piperacillin/tazobactam for severe, non-purulent ABSSSIs [36]. Treatment of purulent ABSSSIs should cover MRSA empirically with doxycycline or trimethoprim/sulfamethoxazole in moderate cases and vancomycin, daptomycin, linezolid, or ceftaroline in severe cases. With the increase of clinical MRSA isolates with decreased susceptibility or resistance to these drugs, treatment of ABSSSIs, especially SSIs, is now challenged by antibiotic resistance, toxicity, few oral options, and greater need for hospitalization and its associated costs [37]. Dalbavancin, a recent addition to the antimicrobial armamentarium that could meet these challenges, is a novel lipoglycopeptide approved by the FDA in May 2014 and by the European Medicines Agency (EMA) in February 2015 for ABSSSIs caused by susceptible Gram-positive organisms [38].

Antimicrobials available in Italy for treating ABSSSIs with activity against MRSA and other resistant Gram-positive pathogens include vancomycin, teicoplanin, daptomycin, linezolid, and ceftaroline. Of the antimicrobials listed, only linezolid has an oral formulation, and some have significant potential for toxicity, including renal impairment from vancomycin, bone marrow suppression, and drug interactions (e.g., selective serotonin re-uptake inhibitors) from linezolid, and myopathy from daptomycin. While doxycycline and trimethoprim/sulfamethoxazole have MRSA activity, their activity against beta-hemolytic streptococci is poorly, limiting their use as monotherapy for ABSSSIs. Dalbavancin is a recently approved, novel lipoglycopeptide which could be an important addition to the antimicrobial armamentarium. It has well-established activity against the Gram-positive organisms commonly involved in superficial and deep SSIs, including MRSA and other multidrug-resistant pathogens, and the MIC_90_ values for these organisms have remained stable over the past decade. Dalbavancin’s high protein binding and prolonged half-life allow for easily and consistently attainable therapeutic levels. Several clinical trials have demonstrated its tolerability, efficacy, and non-inferiority compared to standard therapy for ABSSSIs. The DISCOVER 1 and DISCOVER 2 studies showed that once-weekly intravenous dalbavancin was not inferior to twice-daily intravenous vancomycin followed by oral linezolid for the treatment of ABSSSIs. Adverse events were reported less frequently in patients treated with dalbavancin than in those treated with vancomycin–linezolid [39]. Indeed, dalbavancin’s most unique feature is its once weekly dosing; thus far it had been approved as a 1000 mg dose followed by 500 mg 1 week later.

Dalbavancin is also approved by the EMA for a 1500 mg, one-shot, single dose [40, 41].

Factors promoting its use as an anti-MRSA treatment are its bactericidal activity, partial activity on biofilm increased by combination with rifampin, no interactions and adverse events and the possibility of an early discharge.

The main characteristics of anti-MRSA drugs used for SSIs are reported in Table 7.

**Table 7:** The main characteristics of anti MRSA drugs used for SSIs

Antibiotic	Battericidal activity/Pharmacodynamic/anti-biofilm activity	Route of administration	Doses	Adverse events	Interaction	Cost (70 kg)
Teicoplanin	Bactericidal with low MIC/time dependent/none	iv im	7-10 mg/Kg die, loading dose	Renal toxicicty	None	50–70 E/die
Vancomycin	Bactericidal with low MIC/time dependent/none	Iv	1 g x 2 die 500 mg x 4 die	Renal toxicicty	Other nephrotoxic drugs	5 E/die
Daptomycin	Bactericidal/concentration dependent/yes	Iv	4–6 mg/kg	Tossicità muscolare	Statine	80–120 E/die
Linezolid	Bacteriostatic/time dependent/none	Iv/oral	1200 mg die	Bome marrow toxicity, neuropathy, serotoninergic syndrome	SSRI	120 E/die
Tigecycline	Bacteriostatic/time dependent/partial	Iv	50 mg x 2 die; 100 mg loading dose	Nausea, vomit, pancreatitis	None	120 E/die
Ceftaroline	Bactericidal/time dependent/none	Iv	600 mg x 2 die	rash	None	96 E/die
Dalbavancin	Bactericidal/concentration dependent/partial (in association with rifampin)	Iv	1000 mg giorno 1, 500 mg giorno 7	No	None	NA
Cotrimoxazole	Bactericidal/time dependent/none	Iv, oral	800/160 mg 3 times a dai	anemia	None	15 E/die
Rifampin	Bactericidal/time dependent/yes	Iv/oral	600 mg once a day	Liver toxicity	Several	6 E/die

## Methods

Three of us (GS, CT and SC) analyzed the literature on the field of SSIs (randomized clinical trials, case–control studies, recommendations and clinical cases) and proposed some major and minor risk factors for SSIs to a board of general and specialist surgeons. We used the Delphi method [42–44] to make operative decisions through different steps of convergence, depending on the consensus reached in each round of consultation of the board.

The board was composed of one vascular surgeon, one orthopedic surgeon, one thoracic surgeon, six general and emergency surgeons and one infectious diseases specialist.

## Results

We identified the major and minor risk factors for MRSA SSIs using the Delphi method. The level of consensus acceptable for approval by the group was more than 80 %. Beside the risk factors we also identified protective factors for these kind of infections.

Table 8 reports the risk factors with rates of agreement in the Delphi consultation.

**Table 8:** Risk factors and protective factors according to italian literature

Risk factors	Marchi et al. OR	Petrosillo et al. OR	Fiorio et al. OR
Duration of surgery > 75 percentile (minor criteria)	1,52	--	--
Hospitalization before major surgery	>48 h 1,22	>24 h 1,45	>48 h statistically significant
Urgent surgical intervention	1,29	1,73	--
ASA ≥ 3 (minor criteria)	1,71	--	--
Age > 70 years (major criteria)	--	1,5	--
Drainage > 3 days	--	2,17	--
NNIS score 2-3	--	3,34	--
diabetes (minor criteria)	--	--	statistically significant
Obesity (minor criteria)	--	--	statistically significant
Protective factors			
Laparoscopy	0,49	--	--
SSIs hospital with active surveillance for at least 2 years	0,71	--	--
Adequate prophylaxis	--	0,68	--

These factors should be validated in prospective studies, in order to refine the identification of patients at higher risk of developing MRSA SSIs or to identify patients with a SSI due to MRSA earlier so that effective antibiotic therapy can be administered promptly.

## Discussion

*S. aureus* is consistently the leading cause of nosocomial infections, including SSIs, and the incidence of MRSA strains is rising dramatically. Admissions to hospital because of MRSA infections have more than doubled in the past decade, and MRSA is the leading cause of SSIs in many academic and community hospitals [45].

MRSA as a cause of a SSI was also shown to be a significant independent risk factor for adverse economic outcomes. The risk-adjusted attributable increase in duration of hospitalization was approximately 1 day and the increase in hospitalization costs was over $1,000. Other studies support the finding that patients with MRSA infections require more health care resources than patients with methicillin-sensitive *S. aureus* (MSSA) infections [46]. Kirkland et al. estimated that the excess hospital costs associated with MRSA SSIs ranged from $3,089 to $35,367 [2]. Engemann et al. found that, among surgical patients, those with MRSA infections were hospitalized 5 days longer than those with MSSA [47]. Hospital charges for patients with MRSA infections were also $62,908 greater than those for patients without an infection and $40,000 greater than for patients with MSSA infections.

Weigelt et al. found that significant independent risk factors increasing cost and duration of hospitalization SSIs due to MRSA included illness severity, transfer from another health care facility, previous admissions (within the preceding 30 days), and other polymicrobial infections (*p <* 0.05) [48].

Because universal, rapid MRSA screening is not recommended by all authors, identifying patients at risk of colonization by MRSA and subsequently of SSIs could be very useful.

Harbarth et al. found that emergency surgery, presence of comorbid condition, immunosuppressive therapy, contaminated surgery, and a surgical duration longer than the 75th percentile were the strongest risk factors for MRSA colonization in surgical patients and these factors were confirmed in a validation cohort. [49] We also tried to identify risk factors for colonization and/or infection by MRSA. We analyzed the literature in order to list and quantify the risks of colonization and/or infection by MRSA in surgical patients. The risk factors and protective factors that emerged from Italian literature are presented in Table 9.

**Table 9:** Risk factors with the rate of agreement at the Delphy method

Major risk factors	Rate of agreement
Surgery with elevate risk of MRSA infection	100 %
SSIs with signs of systemic infections	100 %
Patients from long term care facilities	100 %
Previous hospitalization (less than 30 days)	89 %
Charlson score > 5 points	100 %
COPD and thoracic surgery	100 %
Antibiotic therapy with beta-lactams (especially cefalosporins and carbapenem) and/or quinolones in the previous 30 days	89 %
Current lengt of stay > 16 days	100 %
Age 75 years or older	100 %
Surgery with prothesis implantation	100 %
Minor risk factors	
Not compensated diabetes or complicated diabetes	100 %
Body mass index > 30	100 %
Previous hospitalization (>30 days, < 12 months)	100 %
Steroids (prednisone 15 mg/die or equivalent dose for at least 15 days)	100 %
Other immunosoppressive therapy	100 %
Renal insufficiency	100 %
COPD not in thoracic surgery	100 %
Antibiotic therapy not with beta-lactams and/or quinolones in the previous 30 days	89 %
Lenght of surgery > 75 percentile (for surgical procedures that last more than 4 h)	100 %
External biliary drainage	100 %
Protective factors	
Hospital with surveillance system of SSIs	100 %
Correct antibiotic prophylaxis	100 %
Laparoscopic surgery	100 %

An algorithmic method may be useful for identifying patients without risk factors (high negative predictive model) but could be less useful for identifying true positive cases (low positive predictive value). We, therefore, tried to assign a different importance to specific risk factors dividing them into major and minor risk factors for MRSA infection in surgical patients in general and in Italy in particular.

After a review of the specific literature, using the Delphi method we attempted to quantify the importance of risk factors and protective factors for MRSA SSIs. This information could be useful for a surgeon in the daily evaluation of patients who have to undergo surgery or patients who are suspected of having a SSI. This instrument is not an alternative to the current, recommended hygiene measures or to adequate, correctly timed antibiotic prophylaxis. In the case of a SSI, after debridement of the wound and culture of the material obtained from the wound, empirical antibiotic therapy should be started, especially if there is a high risk that the pathogenic agent is MRSA. Dalbavancin, with its long half-life (186 h), should also be administered in the case of programmed discharge of the patient. According to the current summary of product characteristics, a second dose is indicated after 7 days. This second administration might also be a useful occasion for the second surgical evaluation of the wound. Because early discharge is almost always recommended for surgical patients, dalbavancin could be a further beneficial tool to treat SSIs correctly, without increasing the duration of hospitalization, especially for those patients who require continued hospitalization only to complete intravenous antibiotic therapy.

## Conclusion

SSIs are still important diseases in the context of ABSSSIs. These infections have a strong impact from both clinical and economic points of view. Therefore, in the pre-operative and post-operative evaluation of patients suspected of developing a SSI, recognizing whether the patient has risk factors for a SSI and especially one caused by MRSA, the most important pathogen in this field, might be very important in the management of surgical patients.

In the case of a SSI, after debridement of the wound and culture of the material obtained from the wound, empirical antibiotic therapy should be started especially if there is a high probability of the pathogenic agent being MRSA. Dalbavancin, with its long half-life (186 h), should also be administered in the case of programmed discharge of the patient. A second dose is indicated after 7 days according to the drug’s summary of product characteristics.

## Abbreviations

SSIs, surgical site infections; MRSA, methicillin-resistant Staphylococcus aureus; S. aureus, staphylococcus aureus; US dollars, United States dollars; DRG, diagnosis related-group; S. epidermidis, staphylococcus epidermidis; CDC, centers for disease control and prevention’s; FDA, food and drug administration; ABSSSIs, acute bacterial skin and skin structure infections; ASA, American Society of anesthesiologists; ICD-9-CM, International classification of diseases, ninth revision, clinical modification; EMA, European medicines agency; MIC_90,_minimum inhibitory concentration_90_; MSSA, methicillin-sensitive S. aureus
